# ApoA1/HDL-C ratio as a predictor for coronary artery disease in patients with type 2 diabetes: a matched case-control study

**DOI:** 10.1186/s12872-024-03986-w

**Published:** 2024-06-24

**Authors:** Farzaneh Ghaemi, Soghra Rabizadeh, Amirhossein Yadegar, Fatemeh Mohammadi, Hassan Asadigandomani, Melika Arab Bafrani, Sahar Karimpour Reyhan, Alireza Esteghamati, Manouchehr Nakhjavani

**Affiliations:** Endocrinology and Metabolism Research Center (EMRC), Vali-Asr Hospital, Tehran University of Medical Sciences, Tehran, Iran

**Keywords:** ApoA1/HDL-C ratio, Apolipoprotein A-I, Apo A1, High-density lipoprotein, Coronary artery disease, Diabetes

## Abstract

**Introduction:**

This study investigated the possible relationship between the Apo lipoprotein A1 /high-density lipoprotein cholesterol (ApoA1/HDL-C) ratio and coronary artery disease (CAD) in patients with type 2 diabetes (T2D).

**Methods:**

This was a matched case-control study of 482 patients with T2D in two groups of CAD and (*n* = 241) non-CAD (*n* = 241). The patients were classified into four quartiles according to the ApoA1/HDL-C ratio, and multivariate logistic regression analysis was performed to assess the relationship between ApoA1/HDL-C and CAD. ROC analysis was also conducted.

**Results:**

This study showed that the ApoA1/HDL-C ratio has an independent association with CAD in individuals with T2D. The CAD group exhibited a significantly higher ApoA1/HDL-C ratio than those without CAD (*p*-value = 0.004). Moreover, the risk of CAD increased significantly across the ApoA1/HDL-C ratio quartiles, with the highest odds in the fourth quartile. The second quartile showed an odds ratio (OR) of 2.03 (*p*-value = 0.048) compared to the first. Moving to the third quartile, the OR increased to 2.23 (*p*-value = 0.023). The highest OR was noted in the fourth, reaching 3.41 (*p*-value = 0.001). Employing a cut-off value of 2.66 and an area under the curve (AUC) of 0.885, the ApoA1/HDL-C ratio predicts CAD among patients with T2D with a sensitivity of 75% and a specificity of 91% (*p*-value < 0.001).

**Conclusion:**

The current study revealed an independent association between ApoA1/HDL-C ratio and CAD in patients with T2D. This ratio can be a promising tool for predicting CAD during the follow-up of patients with T2D, aiding in identifying those at higher risk for CAD.

## Introduction

In recent decades, the prevalence of diabetes has risen, attributed to factors including global aging, economic expansion, rapid urbanization, sedentary lifestyle, and shifts in nutrition [1]. The International Diabetes Federation (IDF) has estimated a rise to 643 million patients with diabetes by 2030 and a further increase to 783 million by 2045 [2]. Diabetes caused 6.7 million deaths in 2021, constituting 12.2% of global deaths [3].

Cardiovascular disease (CVD), including coronary artery disease (CAD), myocardial infarction (MI), stroke, and congestive heart failure, is the primary cause of mortality and morbidity among individuals with diabetes [4–6]. Diabetes is an independent risk factor for CVD [7]. CVD risk factors such as dyslipidemia, hypertension, obesity, and autonomic dysfunction are also more prevalent in patients with diabetes [8, 9]. Timely diagnosis of CVD in patients with diabetes faces challenges due to altered or obscured typical CVD symptoms [10]. Additionally, diabetes often coexists with other conditions that can interfere with diagnostic tests [11]. Moreover, while indicated in certain situations, diagnostic interventions, including cardiac exercise test, cardiac radionuclide scan, computed tomography (CT) angiography, and cardiac catheterization, are costly and come with several associated complications [12]. Consequently, for early diagnosis and screening of high-risk patients, the tendency to use biochemical markers has increased [13, 14].

Earlier studies have consistently shown that increased low-density lipoprotein cholesterol (LDL-C) levels and decreased high-density lipoprotein cholesterol (HDL-C) levels have significant risks for CVD, especially in individuals with T2D [15–17]. HDL-C plays a critical role in preventing and treating atherosclerotic disease. This is attributed to its anti-inflammatory properties and positive impact on reverse cholesterol transport (RCT), a mechanism responsible for eliminating surplus cholesterol from peripheral tissues and transporting it to the liver [18].

ApoA1, as one of the main components of HDL-C, plays a vital role in the formation of most plasma-esterified cholesterol and helps to promote cholesterol efflux from tissues to the liver by being a cofactor for lecithin cholesterol acyl transferase (LCAT) [19, 20]. ApoA1 modulates LDL-C function and its clearance by the liver. ApoA1 reduces the adverse effects of LDL-C on intravascular atherosclerotic plaque formation and endothelial cell damage. Furthermore, ApoA1 provides protection against functional impairment in the islet beta cells [16, 21, 22]. HDL and ApoA1 are cardioprotective [23]. Studies have demonstrated that elevated ApoA1 and HDL-C concentrations are correlated with a reduction in the severity of coronary artery stenosis [24]. In addition, a lower Apo lipoprotein B/ Apo lipoprotein A1 (ApoB/ApoA1) ratio has been linked to a decreased risk of CVD [25]. Moreover, current literature suggests an association between the ApoA1/HDL-C ratio and diabetes [26].

Prior research has investigated the association between biochemical markers and CVD among patients with T2D. Evidence indicates that levels of circulating growth differentiation factor 15 (GDF-15), the ApoB/ApoA1 ratio, high-sensitivity cardiac troponin I (hs-cTnI), N-terminal prohormone of brain natriuretic peptide (NT-proBNP), and high-sensitivity C-reactive protein (hs-CRP) can be used as predictors of CVD in diabetes [13, 27]. In addition, elevated lipoprotein(a) levels in individuals with diabetes are linked to higher ASCVD risk, and adding lipoprotein(a) to traditional risk factors improves ASCVD risk prediction [28]. However, it remains unclear whether the ApoA1/HDL-C ratio is linked to CVD in individuals with diabetes. This study aimed to investigate the correlation between the ApoA1/HDL-C ratio and CVD in patients with T2D and to assess its potential for predicting CVD occurrence.

## Methods

### Study population

**Fig. 1:**
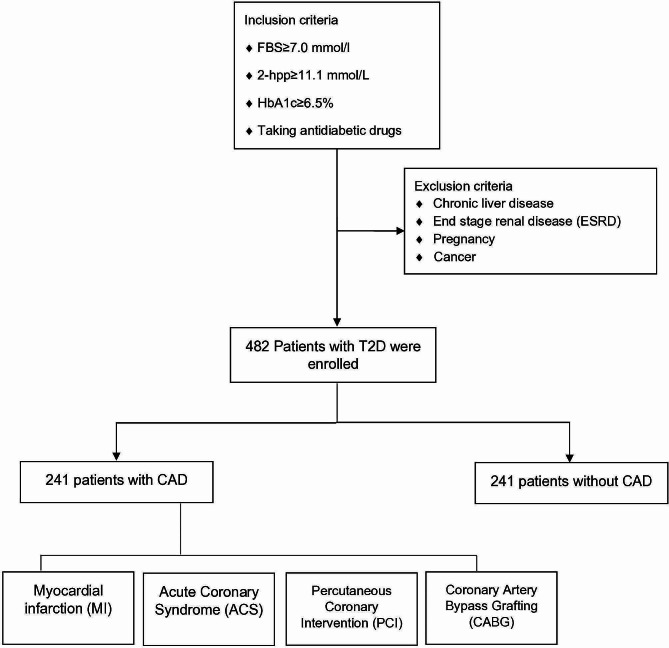
Diagram of patient selection FBS: fast blood sugar; 2-hpp: 2-hour postprandial blood glucose; HbA1c: hemoglobin A1c; T2D: type 2 diabetes; CAD: coronary artery disease

This cross-sectional, matched case-control study was conducted among patients with T2D who attended the diabetes clinic at a tertiary hospital affiliated with Tehran University of Medical Sciences from March 2020 to March 2021. All patients with a diagnosis of T2D according to American Diabetes Association (ADA) criteria [29] and older than 18 years were included in the study. Patients with a history of chronic liver disease, End Stage Renal Disease (ESRD), pregnancy, and cancer were excluded. A total of 482 patients with T2D in two groups of CAD and (*n* = 241) non-CAD (*n* = 241) were enrolled in this study. CAD was defined as the presence of one of the following conditions: history of myocardial infarction (MI), acute coronary syndrome (ACS) leading to hospitalization, Percutaneous Coronary Intervention (PCI), and Coronary Artery Bypass Grafting (CABG) (Fig. 1). The research ethics committee of the Tehran University of Medical Sciences approved the study (IR.TUMS.IKHC.REC.1400.193). The research was conducted in accordance with the Helsinki Declaration. All participants provided informed consent.

### Data collection

A structured questionnaire including age, gender, diabetes duration, history of any disease, history of tobacco and alcohol use, smoking, drug history, history of hospitalization and surgery, and history of coronary artery disease was completed for each patient. Microvascular complications, including retinopathy, neuropathy, and diabetic kidney disease (DKD), were evaluated according to a comprehensive physical exam, laboratory findings, or medical history. Well-trained examiners performed anthropometric measurements, including weight, height, waist circumference (WC), and hip circumference (HC). A calibrated balance beam scale was used to determine the weight (rounded to the nearest 0.1 kg). Using a portable stadiometer, standing height was measured (rounded to the nearest 0.1 cm). This study measured WC at the middle point between the lower borders of the rib cage and the iliac crest (rounded to the nearest 0.1 cm). HC was measured at the widest circumference of the buttock (rounded to the nearest 0.1 cm). To calculate body mass index (BMI), weight was divided by height squared (m^2^). By dividing WC (cm) by HC (cm), the waist-to-hip ratio (WHR) was calculated. Well-trained nurses measured blood pressure (systolic and diastolic) after 10 min of resting in the seated position with a calibrated mercury sphygmomanometer. The definition of hypertension was as follows: systolic blood pressure (SBP) ≥ 140 mm Hg or diastolic blood pressure (DBP) ≥ 90 mm Hg or treatment with antihypertensives, according to ADA guidelines [30]. Blood samples were taken from the patients. The glucose oxidase method was used to determine fasting blood sugar (FBS) and 2-hour postprandial blood glucose (2hpp) (Parsazmun, Karaj, Iran) (Auto Analyzer, BT-3000(plus), Biotechnica, Italy). To measure hemoglobin A1c (HbA1c), high-performance liquid chromatography (HPLC) was employed (DS5, DREW, England). Triglycerides (TG), total cholesterol (TC), LDL-C, HDL-C, and serum creatinine were quantified by enzymatic methods (Parsazmun, Karaj, Iran) (Auto Analyzer, BT-3000(plus), Biotechnica, Italy). Turbidometry was utilized to assess the level of ApoA1. ApoA1/HDL-C ratio was also calculated. The Chronic Kidney Disease Epidemiology Collaboration (CKD-EPI) equation was used to calculate the estimated glomerular filtration rate (eGFR).

### Statistical analysis

Statistical analysis was conducted using Python version 3.12 with NumPy version 1.26, Pandas version 2.1.4, Matplotlib version 3.8.1, and Scikit-learn version 1.4.0 libraries [31–34], and SPSS version 22.0 for Windows (IBM Corporation, New York, USA). Mean and standard deviation (SD) were used to describe continuous variables, while categorical variables were expressed as frequency and percentage (%). The study population was tested for normality using Kolmogorov-Smirnov and Shapiro-Wilk tests, P-P plots, and histograms. As quantitative variables were normally distributed, Student’s t-test analysis was performed to compare the means between different groups. A Mann-Whitney U test was also conducted to assess differences in nonparametric variables such as ‘duration of diabetes.’ The chi-square test and Cochrane’s Mantel-Haenszel statistics were employed to compare categorical variables. A multivariate logistic regression analysis was performed to evaluate the association between ApoA1/HDL-C and other indicators with CAD. The results were expressed as odds ratios (ORs) and a 95% confidence interval (CI). A receiver operating characteristic (ROC) curve was used to estimate the predictive value of ApoA1/HDL-C for the CAD group, and the cut-off for ApoA1/HDL-C was calculated using the maximum Youden index. In this study, a *p*-value < 0.05 was considered statistically significant.

## Results

### Baseline characteristic

In this study, 482 patients with T2D were included, including 241 with CAD and 241 without CAD. The mean age in the CAD group was 61.6 ± 8.1 years, and in the group without CAD was 61.3 ± 8.1years. Approximately 59% of subjects in each group were male. Duration of diabetes, smoking, hypertension, insulin use, and ApoA1/HDL-C ratio was significantly higher in the CAD group than in the group without CAD (*p*-values were < 0.001, < 0.001, 0.006, 0.001, and 0.004, respectively). Using oral agents for diabetes, TC, HDL-C, and LDL-C were significantly lower in the CAD group (*p*-values were 0.001, < 0.001, 0.002, and < 0.001, respectively). Regarding other variables, there was no significant difference between the two groups. Table 1 provides a summary of the baseline characteristics of the study population.

**Table 1:** Comparison of baseline characteristics of the patients with T2D with or without CAD

	T2D without CAD (*n* = 241)	T2D with CAD (*n* = 241)	*p*-value
***Clinical variables***			
Age (years)	61.3 ± 8.1	61.6 ± 8.1	0.652
Male (N, %)	143(59.3)	143(59.3)	-
Duration of diabetes (years)	11 (8–15)	14 (10–20)	< 0.001*
BMI (kg/m^2^)	29.2 ± 4.5	30.0 ± 12.5	0.368
WC (cm)	99.9 ± 10.8	99.9 ± 10.5	0.972
WHR	0.94 ± 0.06	0.94 ± 0.08	0.730
Smoking (N, %)	26(10.8)	150(62.2)	< 0.001*
Hypertension (N, %)	100(41.5)	131(54.4)	0.006*
SBP (mmHg)	129.9 ± 14.6	129.8 ± 16.8	0.958
DBP (mmHg)	79.7 ± 8.3	79.2 ± 7.9	0.513
Microvascular Complications (%)	140(58.1)	158(65.6)	0.111
***Laboratory findings***			
FBS (mg/dl)	153.7 ± 48.5	159.7 ± 53.8	0.203
2-hpp (mg/dl)	220.8 ± 84.4	228.5 ± 85.3	0.318
HbA1c (%)	7.69 ± 1.82	7.89 ± 1.60	0.195
TG (mg/dl)	149(111–199)	145(105-196.5)	0.560
TC (mg/dl)	181.3 ± 42.8	166.4 ± 38.3	< 0.001*
LDL-C (mg/dl)	103.7 ± 34.3	91.7 ± 31.3	< 0.001*
HDL-C (mg/dl)	46.4 ± 13.1	43.1 ± 10.9	0.002*
eGFR (ml/min/1.73 m^2^)	79.7 ± 23.2	77.8 ± 24.9	0.375
ApoA1/HDL-C	2.92 ± 0.78	3.13 ± 0.80	0.004*
***Medication***			
Insulin (N, %)	35(14.5)	66(27.4)	0.001*
Oral agents for diabetes (N, %)	206(85.5)	175(72.6)	0.001*
Anti-lipid drugs (%)	156(64.7)	167(69.3)	0. 272

Data are presented as mean ± SD, median (Q1-Q3), or N (%)

*: *p*-value < 0.05.

T2D: type 2 diabetes; CAD: coronary artery disease; BMI: body mass index; WC: waist circumference; WHR: waist-to-hip ratio; SBP: systolic blood pressure; DBP: diastolic blood pressure; FBS: fast blood sugar; 2-hpp: 2-hour postprandial blood glucose; HbA1c: hemoglobin A1c; TG: triglycerides; TC: total cholesterol; LDL-C: low-density lipoprotein cholesterol; HDL-C: high-density lipoprotein cholesterol; eGFR: estimated glomerular filtration rate; ApoA1: apo lipoprotein A1.

### Association between ApoA1/HDL-C ratio and CAD in patients with T2D

**Fig. 2:**
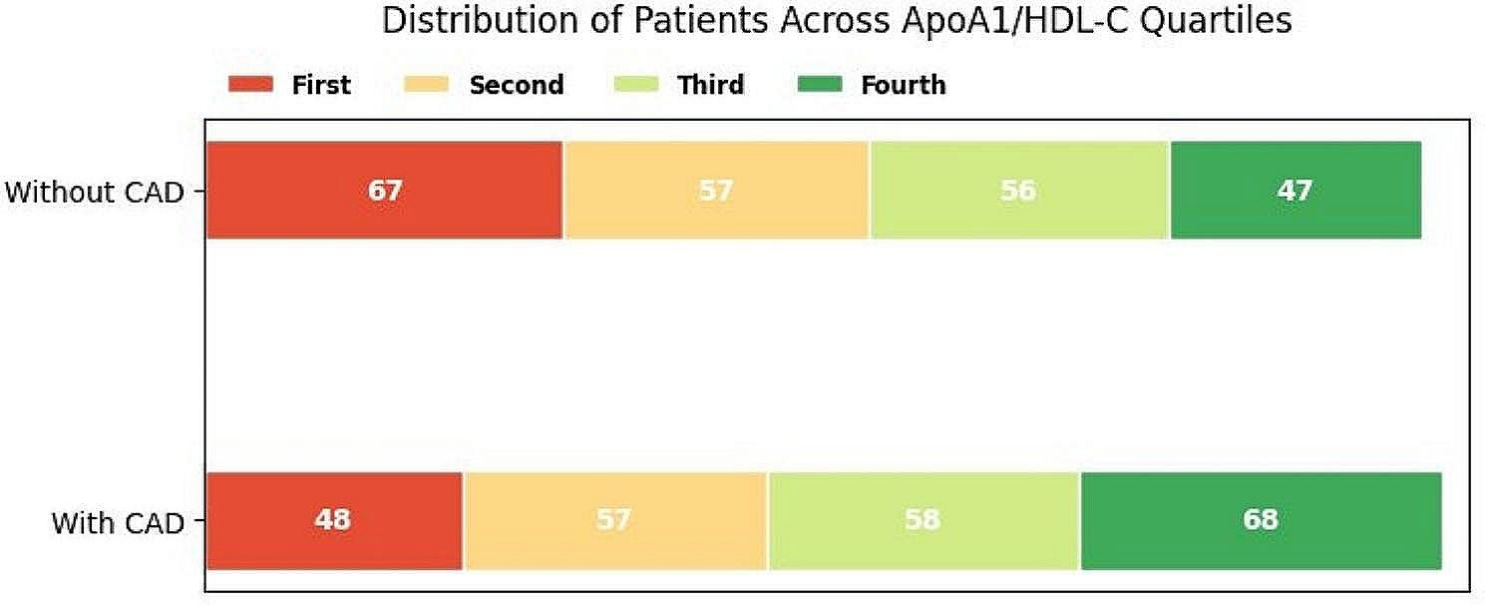
Prevalence of patients in four groups according to ApoA1/HDL-C ratio quartiles

CAD: coronary artery disease; ApoA1: apo lipoprotein A1; HDL-C: high-density lipoprotein cholesterol.

This analysis divided patients into four quartiles to assess the correlation of ApoA1/HDL-C with CAD in patients with T2D. The first quartile included the patients with ApoA1/HDL-C between 0.5 and 2.4. The ApoA1/HDL-C ratio between 2.4 and 3, 3-3.51, and 3.51–5.89 comprised the second to fourth quartile, respectively. As shown in Fig. 2, the first quartile consists of 48 (20%) patients with T2D and CAD, and the fourth quartile includes the highest number of patients (28%). The number of patients in the group without CAD decreased as we moved towards the fourth quartile of the ApoA1/HDL-C ratio. In multivariable logistic regression, after adjusting for confounding factors, including duration of diabetes, HbA1c, smoking, BMI, hypertension, antidiabetic agents, and anti-lipid drugs, the ApoA1/HDL-C ratio was significantly associated with CAD in individuals with T2D.

The first quartile was considered as reference. The second quartile’s OR was 2.03 (95% CI: 1.01–4.10; *p*-value = 0.048). The third quartile had a higher OR, at about 2.23 (95% CI: 1.11–4.46; *p*-value = 0.023), and the fourth quartile had the highest OR, at about 3.41 (95% CI: 1.68–6.91; *p* value = 0.001) (Table 2).

**Table 2:** Association of CAD with ApoA1/HDL-C ratio according to ApoA1/HDL-C quartile in patients with T2D and CAD

ApoA1/HDL-C quartiles (Range)	OR	95% CI	*p*-value
First (0.5–2.4)	1^a^	-	-
Second (2.4–3)	2.03^b^	1.01–4.10	0.048*
Third (3–3.51)	2.23	1.11–4.46	0.023*
Fourth (3.51–5.89)	3.41	1.68–6.91	0.001*

^a^: First quartile was used as reference

^b^: ORs were adjusted for duration of diabetes, HbA1c, smoking, body mass index, hypertension, antidiabetic agents, and anti-lipid drugs

*: *p*-value < 0.05

CAD: coronary artery disease; ApoA1: apo lipoprotein A1; HDL-C: high-density lipoprotein cholesterol; T2D: type 2 diabetes; OR: odds ratio; CI: confidence interval.

### ROC curve analysis

**Fig. 3:**
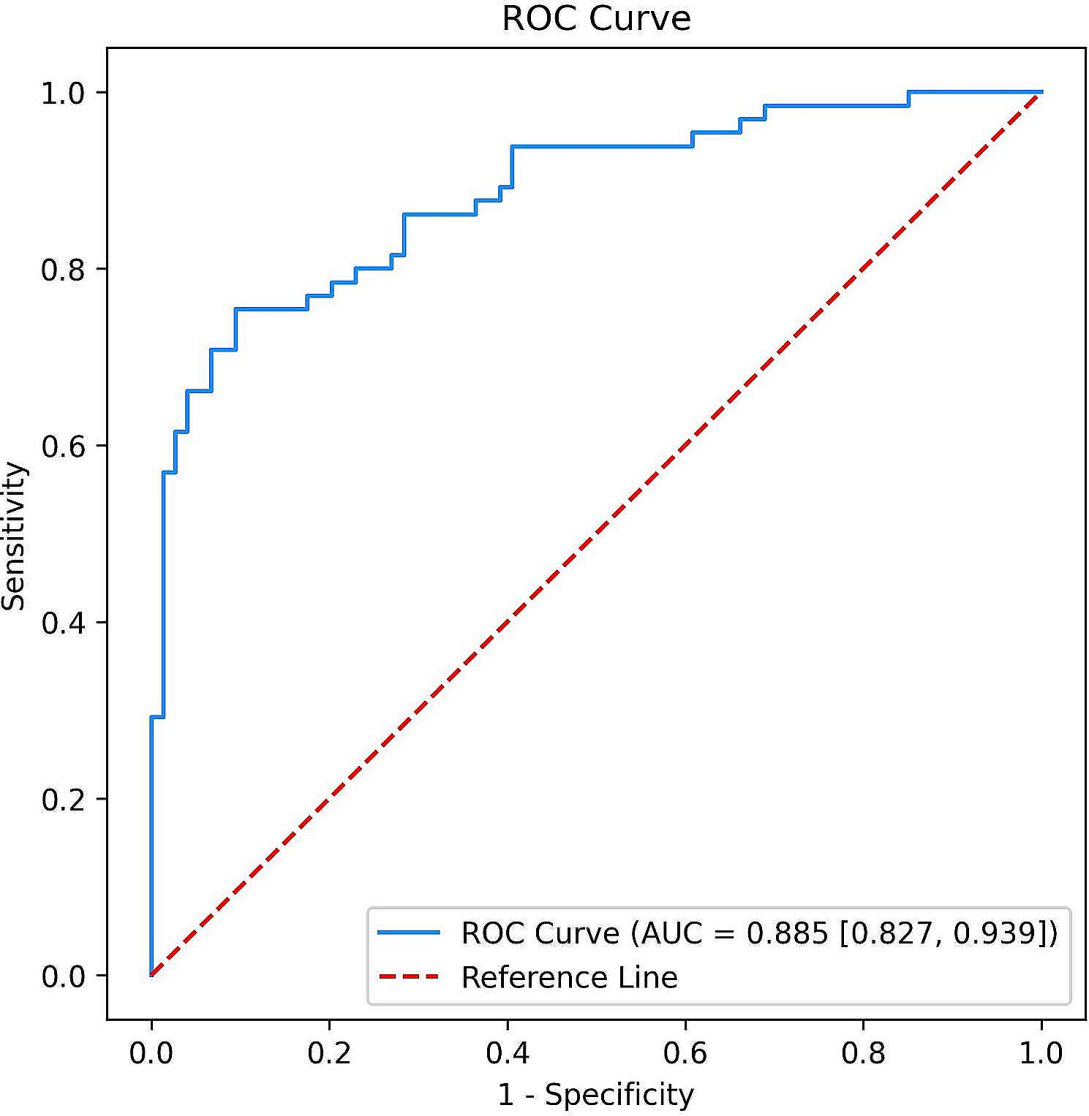
ROC curve, sensitivity, and specificity of ApoA1/HDL-C ratio in diagnosis of CAD in patients with T2D; adjusted for duration of diabetes, HbA1c, smoking, body mass index, hypertension, antidiabetic agents, and anti-lipid drugs ROC: Receiver operating characteristic; ApoA1: apo lipoprotein A1; HDL-C: high-density lipoprotein cholesterol; CAD: coronary artery disease; T2D: type 2 diabetes.

Fig. 3 shows the predictive value of the ApoA1/HDL-C ratio for CAD in patients with T2D based on ROC analysis. Using the maximum Youden Index, the cut-off was set at 2.66 with 75% sensitivity and 91% specificity (AUC = 0.885, 95% CI = 0.827–0.939, *p*-value < 0.001) (Table 3).

**Table 3:** Characteristics of the value of the area under the curve of ApoA1/HDL-C ratio in patients with T2D and CAD

AUC ^a^	Sensitivity	Specificity	95% CI	Cut-off	*p*-value
0.885	75%	91%	0.827–0.939	2.66	< 0.001*

^a^: Adjusted for duration of diabetes, HbA1c, smoking, body mass index, hypertension, antidiabetic agents, and anti-lipid drugs.

*: *p*-value < 0.05.

ApoA1: apo lipoprotein A1; HDL-C: high-density lipoprotein cholesterol; T2D: type 2 diabetes; CAD: coronary artery disease; AUC: area under the curve; CI: confidence interval.

## Discussion

This study investigated the association between ApoA1/HDL-C ratio and CAD in patients with T2D, aiming to clarify whether this ratio has predictive value for CAD in diabetes. The ApoA1/HDL-C ratio was significantly higher in the CAD group than in those without CAD. The current analysis showed that the ApoA1/HDL-C ratio has an independent association with CAD in patients with T2D. Furthermore, the odds ratio for CAD increased across the ApoA1/HDL-C ratio quartiles, with the fourth quartile having the highest OR. With a cut-off of 2.66 and AUC of 0.885, the ApoA1/HDL-C ratio can predict CAD in patients with T2D with a sensitivity of 75% and a specificity of 91%.

Prompt diagnosis of CVD in patients with T2D is vital, as CVD ranks first in terms of cause of death in these patients [5]. Recent studies have investigated the role of biomarkers in predicting CVD in patients with T2D. Mei et al. surveyed patients with T2D with or without CAD and showed that serum GDF-15 levels and ApoB/ApoA1 ratio were higher in the CAD group. Furthermore, CAD was positively correlated with serum GDF-15 or ApoB/ApoA1 ratio. They suggested that these markers may help predict the occurrence of CAD in patients with T2D [13]. In another study, Haller et al. found that hs-cTnI, NT-proBNP, and hs-CRP were independently associated with cardiovascular events and can improve cardiovascular risk prediction [27]. To the best of our knowledge, this study is the first to investigate the association between the ApoA1/HDL-C ratio and CAD in patients with T2D. A cross-sectional survey by Jian et al. investigated the relationship between the ApoA1/HDL-C ratio and diabetes. It showed that an increase in the ApoA1/HDL-C ratio was associated with the incidence of diabetes and was a primary risk factor for diabetes in both genders [26]. In addition, a case-control study by Nakhjavani et al. showed that a higher ApoA1/ HDL-C ratio was correlated with microalbuminuria in females with T2D [35]. However, the association of this ratio with CAD in patients with T2D has yet to be reported. The independent association between ApoA1/HDL-C ratio and CAD that this study showed not only helps predict CAD but can also help elucidate the possible underlying pathways of CAD in patients with T2D, which further studies can investigate.

Several studies have shown that HDL-C and its major component, ApoA1, are cardioprotective [23]. A study in the general population found that ApoA1 levels were lower among patients with CAD, and low ApoA1 levels were independently associated with CAD presence and severity [24]. Another study compared ApoA1 and other lipid parameters in three groups of patients, including those with CAD but without T2D, patients with CAD and T2D, and a control group. Researchers found significantly lower ApoA1 levels in the CAD-positive group than in the control group [36]. In addition, studies showed that the patients with atrial fibrillation had significantly lower ApoA1 levels than the control group [37, 38]. A case-control study compared the ApoA1 levels in patients with diabetes and non-diabetic healthy individuals. They demonstrated that the mean ApoA1 levels were lower in patients with diabetes compared to the healthy participants [39]. In a meta-analysis of patients with or without diabetes treated with statins, an increase in HDL-C levels after treatment had no significant cardiovascular benefit, whereas an increase in ApoA1 resulted in a substantial reduction in cardiovascular events [40]. In the present study, the ApoA1/HDL-C ratio was significantly higher in the CAD group, and the odds ratio for CAD was greater in the higher quartiles of the ApoA1/HDL-C ratio compared to the first quartile. Although HDL-C levels are critical, recent studies indicate its function holds even greater importance. It has been demonstrated that the simple measurement of HDL-C concentration is not always associated with cardiovascular risk [41]. HDL-C can be influenced by biomarkers such as 15-lipoxygenase (15-LPO), myeloperoxidase (MPO), symmetric dimethylarginine (SDMA), and other markers. These influences alter HDL-C, decreasing the availability of endothelial nitric oxide, causing problems in endothelial repair, triggering pro-inflammatory activation, and leading to macrophage efflux, ultimately affecting its function [18]. Huang et al. illustrated that HDL-C and ApoA1, obtained from human atheroma, were dysfunctional and oxidized by MPO [42]. Moreover, in vitro studies revealed that oxidized ApoA1 and HDL-C particles reduce their ability for optimal cholesterol acceptance and make these particles poor in lipid content. As a result, they could behave as pro-inflammatory molecules and initiate the process of augmented atherogenesis and increased risk of CVD [42–45]. Studies illustrated that the function and structure of HDL-C change in patients with diabetes [46]. Additionally, evidence suggests that ApoA1 is a more reliable and stable marker than HDL-C [47]. Therefore, the ApoA1/HDL-C ratio may better represent HDL-C structure and function and better predict CVD in patients with diabetes.

The ApoA1/HDL-C ratio’s predictive accuracy for CAD in patients with T2D was relatively high in this study (AUC = 0.885), and it holds promise for clinical and epidemiological research. The lower cost and easy accessibility make ApoA1 and HDL-C measurements advantageous over other diagnostic tests, especially invasive procedures. Moreover, given the crucial need for early CAD diagnosis in patients with T2D, ApoA1/HDL-C ratio testing adds significant value.

This study found that the duration of diabetes, smoking, and hypertension were significantly higher, while HDL-C levels were significantly lower in the CAD group. Given that the abovementioned factors are known risk factors for CAD, it is not surprising that they are more prevalent in the CAD group compared to the group without CAD [48, 49]. On the contrary, TC and LDL-C levels were significantly lower in the CAD group. These differences may be due to more severe lipid-lowering therapies in patients with CAD. In addition, patients with CAD may be more adherent to lifestyle and nutritional modifications.

### Strengths and limitations

This study was the first to examine the relationship between ApoA1/HDL-C and CAD in patients with T2D and its role as a predictive factor for CAD. In addition, this was an age- and sex-matched case-control study that minimized potential confounding factors related to age and gender, allowing for a more accurate assessment of the association. Nonetheless, there were some limitations to this study. Firstly, the number of patients was limited. Secondly, the findings of this analysis may not be fully generalizable to the entire diabetic population, as this study was conducted at a single center and included a limited number of participants. Thirdly, due to its case-control nature, this study cannot conclude a causal link between the ApoA1/HDL-C ratio and CAD. Moreover, there is a possibility of the presence of selection bias. Future research could explore additional factors, such as GDF-15, that may influence the ApoA1/HDL-C ratio and its association with CAD in patients with T2D. In addition, further studies could examine the predictive value of the ApoA1/HDL-C ratio for CAD over time through longitudinal and more extensive studies. In future research, the inclusion of a healthy control group could enhance the comprehensiveness of the findings.

## Conclusion

This study showed that the ApoA1/HDL-C ratio has an independent association with CAD in patients with T2D. Incorporating the ApoA1/HDL-C ratio into current risk assessment models may improve their predictive accuracy. Furthermore, ApoA1/HDL-C ratio could assist in the early identification of patients with T2D who are at high risk for CAD, leading to more targeted patient management strategies. The ApoA1/HDL-C ratio holds potential as a valuable tool for clinicians in the risk stratification and personalized patient care.

## Data Availability

The data that support the findings of this study are available on request from the corresponding author.
